# Hormonal and behavioral responses to an infant simulator in women with and without children

**DOI:** 10.1002/dev.22321

**Published:** 2022-09-06

**Authors:** Hanneli Sinisalo, Marian J. Bakermans‐Kranenburg, Mikko J. Peltola

**Affiliations:** ^1^ Human Information Processing Laboratory, Faculty of Social Sciences, Psychology Tampere University Tampere Finland; ^2^ Faculty of Behavioural and Movement Sciences, Educational and Family Studies Vrije Universiteit Amsterdam Amsterdam The Netherlands; ^3^ Tampere Institute for Advanced Study Tampere University Tampere Finland

**Keywords:** caregiving behavior, cortisol, estradiol, fertility motivation, hormonal reactivity, oxytocin, testosterone

## Abstract

We investigated the impact of maternal status on hormonal reactivity and behavioral responses to an infant simulator in 117 women (54 primiparous, 63 nulliparous). The amount of affectionate touch and motherese were analyzed as behavioral measures of caregiving. Saliva was collected before and 10 min after interaction with the infant simulator to analyze oxytocin, testosterone, cortisol, and estradiol levels. Nulliparous women also provided information about their fertility motivation. Linear mixed models indicated that greater use of affectionate touch was associated with lower overall testosterone levels. Cortisol decreased in response to the interaction in both groups. In the primiparous group, the amount of affectionate touch associated inversely with cortisol levels, whereas in the nulliparous group such association was not found. Oxytocin or estradiol reactivity to the simulator did not differ between the groups, nor were these hormones associated with behavior. Higher fertility motivation in nulliparous women was related to more motherese, and lower testosterone levels. Our results indicate that the simulator elicits hormonal reactivity both in mothers and nonmothers, but the patterns of associations between caregiving behavior and hormonal levels may be partially different. These results encourage using the infant simulator to explore hormonal processes related to the transition to parenthood.

## 1 INTRODUCTION

The transition to parenthood in females is associated with major biological and behavioral changes. In animals, the biological changes associated with reproduction are well established (Brunton & Russell, 2008; Rilling, 2013), but in humans these changes are less well documented. Some earlier studies have indicated that gray matter volume decreases during pregnancy in human mothers (Hoekzema et al., 2017; Kim et al., 2010; Oatridge et al., 2002), and these changes are observed in areas important for social cognition, empathy, and emotion regulation (Adolphs, 2009; Kim, 2016; Rocchetti et al., 2014), which are all essential for maternal behavior and sensitivity to infant signals. In both women and men, parents and nonparents have also been shown to differ in their brain activity in response to infant cues (Nishitani et al., 2011; Peltola et al., 2014; Proverbio et al., 2006; Seifritz et al., 2003; Witteman et al., 2019). In addition to changes in brain structure and functioning, hormonal changes during pregnancy have been found in both mothers and fathers (Edelstein et al., 2015). Prenatal changes in hormonal levels may be important for postnatal parenting, as key parenting‐related hormones such as oxytocin and testosterone have been associated, for example, with attachment (Strathearn et al., 2009), attraction toward infants (Fleming, Steiner, et al., 1997), and parental sensitivity (Feldman et al., 2007; Glynn et al., 2016). Currently, there are no studies comparing caretaking behavior between new parents and nonparents or examining whether caretaking behavior is similarly associated with hormone levels in these two groups. Comparing parents and nonparents directly is important for understanding whether hormonal influences on caretaking behavior (or vice versa) might change across the transition to parenthood. In this study, we examine the behavioral correlates of oxytocin, testosterone, cortisol, and estradiol in primiparous and nulliparous women while they are in interaction with an infant simulator.

### 1.1 Oxytocin

The neuropeptide oxytocin is produced and segregated in the hypothalamus (Brunton & Russell, 2008) and it is essential for contractions during labor (Blanks & Thornton, 2003) and lactation (Augustine et al., 2018). Animal studies indicate that oxytocin is critical for the onset of maternal behavior (Rilling & Young, 2014). For example, in rat dams, oxytocin augments approach motivation toward pups through the dopamine network, strengthening maternal behavior (Rilling, 2013).

In humans, plasma oxytocin levels may increase already when women are in a relationship (Schneiderman et al., 2012). Levine et al. (2007) observed that during pregnancy, some mothers displayed an increase in their plasma oxytocin levels, whereas others showed decreasing or constant levels throughout pregnancy. Increasing oxytocin levels during pregnancy also correlated with self‐reported prenatal bonding with the fetus (Levine et al., 2007). After pregnancy, both maternal and paternal oxytocin levels increased similarly during the first six postnatal months (Gordon et al., 2010). Higher oxytocin levels in maternal saliva have been associated with more positive behavior toward their 4‐ to 6‐month‐old infants (e.g., gaze toward infant, positive affect, and infant‐directed speech) and higher mother–infant interaction synchrony (Feldman et al., 2011). Higher maternal plasma oxytocin levels have also been associated with more affectionate touch and eye contact when mothers engage in face‐to‐face interaction with their infants (Feldman et al., 2012). Similarly, Gordon et al. (2010) noticed that higher maternal plasma oxytocin levels were associated with affectionate parenting behaviors (“motherese” vocalizations, expression of positive affect, and affectionate touch) when assessed during the first six postpartum months. No studies, however, have directly compared oxytocin levels in parents and nonparents or investigated whether peripheral oxytocin levels are associated with behavior toward infants also in nonparents. Investigating associations between oxytocin and maternal behavior in both mothers and nonmothers is important for a better understanding of whether oxytocin may be associated with caretaking behavior already before the transition to parenthood.

### 1.2 Estradiol

Together with progesterone, the ovarian steroid estradiol prepares the uterus for pregnancy and estradiol levels increase throughout pregnancy, peaking just before birth (Edelstein et al., 2015; Fleming, Ruble, et al., 1997; Glynn et al., 2016). Estradiol levels decrease rapidly in the postnatal period in women (Fleming, Ruble, et al., 1997). In addition to reproductive functions, estradiol is associated with individual differences in responses to emotional intimacy (Edelstein et al., 2010), but the effects of estradiol on parent–infant interaction or maternal behavior have been very little studied. One study observed that mothers with lower estradiol increase during pregnancy showed more sensitive parenting behavior 1‐year postpartum (Glynn et al., 2016). Relatedly, women with a lower prenatal estradiol increase were rated as providing more spousal support by their partner (Edelstein et al., 2017). The mechanisms underlying the somewhat paradoxical negative relations between prenatal estradiol levels and postnatal parenting outcomes are not clear, but it is possible that they are related to interactions between estradiol and other hormones such as testosterone (Bakermans‐Kranenburg et al., 2022). However, the preliminary findings are partially in line with studies on nonhuman primates, which have associated higher estradiol levels during pregnancy with less optimal maternal behavior toward offspring (Fite & French, 2000; French et al., 2004).

### 1.3 Cortisol

Having an important role in fetal maturation, maternal cortisol levels increase during pregnancy, remain elevated for the first postnatal weeks, and then decline to their original levels both in plasma (Fleming, Steiner, et al., 1997) and urine samples (Conde & Figueiredo, 2014). Furthermore, cortisol levels are higher in primiparous than multiparous women (Bleker et al., 2017; Conde & Figueiredo, 2014) and this difference is partly mediated by pregnancy‐specific distress in primiparas who tend to experience more stress symptoms through pregnancy than multiparas (Gillespie et al., 2018).

Higher salivary cortisol levels have been linked to higher sensibility to the own infant's body odor (Fleming, Steiner, et al., 1997) and higher self‐reported sympathy to crying infant stimuli in postpartum women (Stallings et al., 2001). On the other hand, higher salivary cortisol levels, measured both cross‐sectionally and longitudinally, have been associated with lower maternal sensitivity (Finegood et al., 2016; Gonzalez et al., 2012). In addition, lower salivary cortisol levels have been associated with less intrusive parenting behavior in mothers of 6‐month‐old infants (Mills‐Koonce et al., 2009). Thus, while elevated cortisol may have a role in a more vigilant response to infant cues, it is negatively associated with observed sensitive parental behavior, likely reflecting the effects of stress on parenting.

### 1.4 Testosterone

Testosterone research has mainly focused on men, with results showing decreasing testosterone levels during the transition to fatherhood. However, testosterone has an important role in female parenting as well, although the associations between testosterone and parenting might be different in women. According to the Challenge hypothesis, decreased testosterone levels facilitate investment in family life and sensitive parenting behaviors in men (Archer, 2006; Gettler, et al., 2011; Meijer et al., 2019). In line with these findings, higher testosterone levels have been associated with lower reproductive ambition in young women (Deady et al., 2006). Testosterone levels change during the prenatal period, but in a sexually dimorphic manner: testosterone levels increase during pregnancy in mothers and decrease in fathers (Edelstein et al., 2015). This suggests that results from male samples cannot be generalized to women.

Postnatally, both fathers and mothers have lower testosterone levels in general than nonparents (Barrett et al., 2013; Fleming et al., 2002; Grebe et al., 2019; Meijer et al., 2019) and lower testosterone levels are associated with better relationship quality in both men and women (Edelstein et al., 2017). In line with the Challenge hypothesis, Weisman et al. (2014) found that higher baseline testosterone is associated with lower paternal sensitivity (gaze, touch, infant‐directed speech). Similarly, Fleming et al. (2002) observed that both fathers and nonfathers with lower testosterone levels expressed a higher need to respond to infant cries than men with higher testosterone levels. In women, higher salivary testosterone levels have been associated with their motivation to view cute infant faces (Hahn et al., 2015). Furthermore, the dual‐hormone hypothesis suggests that the association of testosterone with behavior depends on cortisol levels: higher cortisol levels may inhibit the effects of testosterone on aggression or dominance (Mehta & Josephs, 2010). The same effect can be found in men's self‐reported empathy: when basal cortisol is low, high testosterone levels predict lower empathy (Zilioli et al., 2015). However, the moderating effects of cortisol on testosterone have so far been studied mainly in men (but see Voorthuis et al., 2019).

### 1.5 Hormonal reactivity

Research on hormonal associations with parenting has largely focused on hormonal baseline levels. Nonetheless, hormonal levels also show short‐term reactivity and the associations of such reactivity with parenting behavior are an important research target. Trajectories of hormonal baseline levels through pregnancy are well established but whether short‐term hormonal reactivity to infant stimuli changes across the transition to parenthood is not known. Hormonal reactivity (measured from saliva or plasma) has been observed in response to various triggers, such as exercise (Copeland et al., 2002; de Jong et al., 2015), stressful situations (Cox et al., 2015; de Jong et al., 2015), massage (Carter et al., 2007; Riem et al., 2017), and breastfeeding (Grewen et al., 2010). Importantly, hormonal reactivity also occurs in response to infant stimuli. For example, fathers’ salivary cortisol levels decreased after interacting with their children (Gettler et al., 2011) and listening to infant cry sounds has been associated with increases in oxytocin and cortisol levels in mothers (Swain et al., 2011). In addition to responses to infant crying, parent–infant touch is a significant aspect of parent–infant interaction. Skin‐to‐skin contact has been found to increase oxytocin levels and decrease cortisol levels both in infants and parents (Cong et al., 2015; Vittner et al., 2018).

Hormonal reactivity has also been found to relate to parental behavior. Kohlhoff et al. (2017) observed that in some mothers oxytocin levels increased while in others oxytocin levels decreased or remained constant when mothers and their 3‐ to 4‐month‐old infants were presented with the still‐face paradigm. Increasing oxytocin levels were associated with higher observed maternal sensitivity. Increased maternal oxytocin levels after mother–infant interaction have also been associated with more affectionate touch (Feldman et al., 2010) and longer duration of mother‐to‐infant gaze (Kim et al., 2014). In addition, oxytocin responses to interaction with the infant have been found to be larger in mothers with secure attachment representations as measured with the Adult Attachment Interview (Strathearn et al., 2009). In single women, attenuated estradiol reactivity to an emotionally intimate parent–infant video was linked to avoidant attachment style (Edelstein et al., 2012). In fathers, testosterone levels decreased during the Strange Situation Procedure and the amount of decrease predicted their sensitive parenting behavior when in interaction with their own 12‐month‐old child (Kuo et al., 2016). Lotz et al. (2022) found associations at trend level between fathers’ sensitivity and both oxytocin reactivity and testosterone reactivity, with more sensitive parenting behavior during a 10‐min interaction with their own 2‐month‐old infant related to stronger increases in fathers’ oxytocin and testosterone levels.

The impact of parental status on hormonal reactivity to infant stimuli has not yet been studied. A few studies have used an infant simulator, which provides a validated and ethical method for studying both parents and nonparents in a realistic caretaking situation (Bakermans‐Kranenburg et al., 2015; Voorthuis et al., 2013). The infant simulator is a doll resembling a real infant in terms of weight, appearance, and affective vocalizations. Testosterone levels in nulliparous women were found to decrease after taking care of a crying infant simulator for 30 min (Voorthuis et al., 2019). A similar effect was observed in men when they interacted with a crying but soothable infant simulator: salivary testosterone levels decreased (van Anders et al., 2012). However, this finding did not replicate in an independent sample: instead of a decrease, testosterone levels were found to stay constant when men interacted with the crying simulator (van Anders et al., 2014). Another study, on the other hand, observed increased testosterone and cortisol levels in pregnant women following interaction with an unsoothable infant simulator (Bos et al., 2018). Postnatally, when the same participants interacted with their own infant, cortisol levels were found to decrease. Thus, cortisol reactivity may be different before and after birth or different to the mother's own infant as compared to an infant simulator. However, neither testosterone nor cortisol was related to observed parental sensitivity in mothers, whereas in fathers decreases both in testosterone and cortisol in response to interaction were related to more sensitive parental behavior (Bos et al., 2018). The associations between hormones (and their reactivity) and caretaking behavior seem to be somewhat different in mothers and fathers, and also in parents and nonparents, which makes it important to study these groups separately.

### 1.6 The present study

In this study, we investigated the impact of maternal status on salivary hormonal reactivity and behavioral responses to an infant simulator. Mothers and nonmothers have not been compared on these outcomes and, therefore, the current study will provide important data for understanding whether the associations between hormones and behavior are independent of parental status. Primiparous and nulliparous women took part in a realistic caretaking situation with an infant simulator that made both positive and negative sounds. Maternal behavior was operationalized as the amount of affectionate touch and motherese vocalizations (i.e., infant‐directed speech) during the caretaking situation. First, we investigated whether the two groups differed in their hormonal reactivity toward the infant simulator. Due to the lack of earlier studies comparing hormonal and behavioral responses toward infants in parents and nonparents, we were cautious in making strong directional hypotheses. Oxytocin reactivity has not been investigated with an infant simulator, but based on research in mothers with their own infants, oxytocin levels were expected to rise at least in the primiparous group (Cong et al., 2015; Swain et al., 2011; Vittner et al., 2018). Considering estradiol, we had a more exploratory approach and did not make directional hypotheses. Based on the available evidence, we predicted that in nulliparous women testosterone levels would decrease during the interaction (Voorthuis et al., 2019), whereas in primiparous women testosterone levels were not expected to show any reactivity, in line with the findings of Bos et al. (2018). Also, according to Bos et al. (2018), one might expect differences in cortisol responses in the two groups, with a decrease in primiparous women and an increase in nulliparous women. However, as that study differed in terms of study population and simulator paradigm from the current study (i.e., pregnant women interacting with a persistently crying simulator), we did not make strong directional hypotheses regarding cortisol. Second, we investigated whether hormonal reactivity is associated with behavior toward the infant simulator. We hypothesized that positive oxytocin reactivity would be associated with greater amount of affective touch and motherese vocalizations (Feldman et al., 2010; Kim et al., 2014; Kohlhoff et al., 2017). Regarding estradiol, testosterone, and cortisol, we did not have clear a priori hypotheses due to insufficient earlier research. Based on earlier research on the dual‐hormone hypothesis (Mehta & Josephs, 2010), we also analyzed whether the association between testosterone and maternal behavior was dependent on baseline cortisol levels. Third, we investigated whether the possible associations between hormones and behavior were different in the two groups. Again, we adopted an exploratory approach due to the nonexistent previous research comparing parents and nonparents during interaction with an infant simulator. Finally, in nulliparous women their attitudes and desires toward reproduction (i.e., *fertility motivation*; Brase & Brase, 2012; Deady et al., 2006) might influence their interaction with the simulator or their hormonal reactivity. Thus, we investigated whether in nulliparous women self‐reported fertility motivation was associated with behavior and hormonal levels as well as hormonal reactivity in response to the infant simulator.

## 2 METHODS

### 2.1 Participants

The participants were part of the TransParent project, which investigates changes in processing infant cues during the transition to parenthood. The study protocol was reviewed by the Ethics Committee of the Tampere Region. The participants were 22‐ to 37‐year‐old women from Pirkanmaa area, Finland. Primiparous women were recruited by invitation letters, which were sent based on contact information of local primiparous families obtained from the Digital and Population Data Services Agency. Nulliparous women were recruited through university email lists. Inclusion criteria for all participants were the age of 22–37 years, relationship duration longer than 6 months, living with their partner, and sufficient skills in Finnish. In addition, nulliparous females were required not to have children (own or partner's) and primiparous women were required to have one approximately 6‐month‐old child (age at the time of the laboratory visit: *M* = 7.19, *SD* = 1.48). Of the primiparous women, 55% were married and in the nulliparous group the percent of married participants was 18%. The majority (82%) of primiparous women were breastfeeding their infant at the time of participation. Hormonal birth control (IUD, oral contraceptives, ring, or capsule) was used by 63% of the nulliparous women and by 20% of the primiparous women. In total, 117 participants (54 primiparous, 63 nulliparous) visited the lab. Originally, we based our targeted sample size of 120 participants on the key analyses of the data collected during the laboratory visit, most of which had a 2 × 2 design. For such analyses, the targeted sample size had a power of .80 (with an alpha level of .05) to detect main effects (i.e., group differences) with an effect size of *d* = 0.52 and larger, and two‐way interactions with an effect size of $\eta_{p}^{2}$ = .064 and larger. As a reward for participation, the participants received one movie ticket and course credit if necessary. Descriptive statistics are presented in Table 1.

**TABLE 1:** Descriptive statistics of the nulliparous and primiparous groups

	Nulliparous		Primiparous			
	*n*	*M* (*SD*)	*n*	*M* (*SD*)	*t* (*df*)	*χ* ^2^
Age	63	26.32 (3.44)	54	29.91 (2.96)	5.99 (115)***	
Relationship duration in years	63	4.57 (3.53)	49	6.49 (3.33)	2.95 (110)**	
Married	11	17.5%	27	55.1%		17.42***
Years of education	63	16.3 (1.96)	49	17 (2.34)	1.74 (110)	
Menstrual cycle day	49	20.84 (10.53)	22	22.41 (7.22)	1.73 (72)	
Time of day	63	13:38 (1:42)	54	14:17 (1:53)	1.864 (115)	
Hormonal birth control^a^	40	63.5%	11	20.4%		21.898***
Income^b^						33.975***
<14,999	26	41.9%	2	4.1%		
15,000–29,999	13	21.0%	5	10.2%		
30,000–49,999	15	24.2%	15	30.6%		
50,000–69,999	3	4.8%	15	30.6%		
70,000–89,999	4	6.5%	10	20.4%		
>90,000	1		2	4.1%		

^a^ Use of oral contraceptives, hormonal IUD, contraceptive implant, or contraceptive patch.

^b^ Annual household income in Euros.

** *p* < .01

*** *p* < .001.

### 2.2 Procedure

The participants were called before the laboratory visit. During the call, menstrual cycle and hormonal contraceptive use were screened. Whenever possible, the laboratory visit was scheduled in the luteal phase of the cycle. The participants were asked not to eat or drink in a full hour before the visit. Similarly, breastfeeding mothers were asked to breastfeed their baby 1 h before the visit (time since breastfeeding: *M*_hour_ = 1.37, *SD* = 1.04). These practices aimed at controlling the effects of menstrual cycle (Engel, Klusmann, et al., 2019), contraceptive use (Montoya & Bos, 2017; Van Anders et al., 2014), and breastfeeding (White‐Traut et al., 2009) on hormonal levels. Visits took place between 12:00 p.m. and 6:00 p.m. to control for the diurnal variation of hormone levels (Endendijk et al., 2016; Van Anders, Goldey & Bell, 2014). The whole laboratory session consisted of six different tasks and lasted approximately 75–90 min. The majority of the tasks were computerized tasks measuring physiological, attentional, and motivational responses to infant stimuli. Those data will be reported elsewhere.

At the beginning of the laboratory visit, participants received information about the study, signed an informed consent, and completed a short questionnaire, which was followed by the first saliva sample (T1). Samples were collected with a Salivette (Sarstedt, Nümbrecht, Germany) polypropylene swab. Participants were asked to chew the swab for 1 min and then insert the swab into a polyethylene container without touching the swab with their hands. The swab was initially stored at −20 to −30°C before being transported in dry ice to a liquid nitrogen freezer (−80°C). The samples were analyzed in the Finnish Institute of Occupational Health (Helsinki, Finland).

Before the infant simulator task, the participants performed a short task and had two ECG electrodes attached to their chest. The electrodes were attached to direct attention away from the video camera and to make the physiological measurement feel more realistic to the participants as they were told that the aim of the infant simulator task was to “examine the physiological reactions caused by interaction with the infant simulator.” The infant simulator was a lifelike doll weighing about 5 kg (https://www.renates‐puppenstube.de/en). The doll had a small Bluetooth speaker inside. The sounds of the infant simulator were vocalizations of real infants varying from full‐blown cry to delighted giggling sounds, which were collected from the internet. Interaction with the infant simulator was videotaped so that it was possible to observe the ongoing interaction from a monitor in an adjacent room. During the infant simulator task, the experimenter controlled the infant sounds with a Bluetooth‐connected laptop. The sounds were presented with VLC media player software. The sequence of events was identical for all participants except for the duration of the crying period. The infant simulator cried as long as participants were changing the diaper and soothed right after the task was done. The videos were recorded and used for behavioral analysis.

**FIGURE 1:**
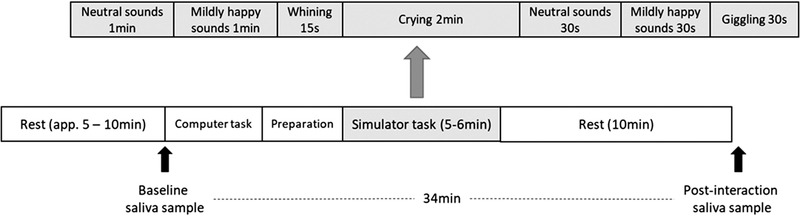
Timeline of the study procedure (below) and timing of infant simulator sounds (above)

The infant simulator was placed in a carrier in the testing room before the participant's arrival so that the participants were able to see the simulator immediately upon entering the laboratory. Before starting the infant simulator task, the experimenter took the simulator to the adjacent room to turn on the speaker and connect it to the laptop for the sound presentation. During this period, the participant completed the 20‐item Positive And Negative Affectivity Schedule (PANAS; Watson et al., 1988) questionnaire that inquired about their current affects. After this, the simulator was returned to the room in the carrier. Participants were given the instruction to spend approximately 6 min (*M* = 5.8 min, *SD* = 0.56 min; Figure 1) with the simulator and try to interact with it like they would interact with a real baby. They also received the instruction to try to soothe the baby if it started to get fussy and that the baby would not calm down without a diaper change if it started to cry very loudly. The testing room had a mat on the floor with various toys, diapers, a changing pad, and wet wipes available. Participants were then left alone with the simulator for approximately 6 min. The simulator made neutral to mildly positive vocalizations during the first 2 min of the task. After 2 min, the simulator started to whimper and soon after cry loudly. The simulator cried in total of 2 min (*M* = 1.86 min, *SD* = 0.38 min). After the participant had changed the diaper, the simulator began to make content vocalizations and eventually giggle for about 30 s. The purpose of this sequence of events was to give the impression of a successful caregiving experience.

After the interaction with the simulator, the participants spent 10 min alone while filling in two questionnaires: one measuring self‐reported empathy (Interpersonal Reactivity Index; Davis, 1983) and the PANAS questionnaire for the second time. After 10 min, the second saliva sample (T2) was collected in the same way as the first sample. The whole procedure is illustrated in Figure 1.

Within days after the laboratory visit, the participant received a link to an online questionnaire, which included questions about background information such as education, income, and relationship status and length. The questionnaire also included items assessing depressive symptoms (CES‐D; Radloff, 1977), anxiety (STAI; Bieling et al., 1998), relationship satisfaction (CSI; Funk & Rogge, 2007), and reflective functioning (RFQ; Fonagy et al., 2016). Primiparous women were also asked about any pregnancy complications, the infant's health, and maternal postnatal attachment representations (MPAS; Condon, 2015). Nulliparous women were asked about their wishes of having children in the future, experience of taking care of children, and their fertility motivation (Attitude Toward Babies Scale [ABS]; Brase & Brase, 2012). All of the nulliparous and 94% of the primiparous participants completed the online questionnaire.

### 2.3 Measures

#### 2.3.1 Behavioral coding from the videos

In this study, we used a frequency‐based method for analyzing behavior, because it is feasible, objective, and suitable for evaluating behavioral responses to predetermined stimuli like an infant simulator. The videos were coded offline with Noldus Observer XT (version 11; https://www.noldus.com/observer‐xt) with a combination of continuous and interval sampling. Using 5‐s intervals, the prevailing behavior of the participant was coded in three different categories that were similar to the categories used in Feldman et al. (2011) and Gordon et al. (2010). *Adult vocalizations* were coded with a 5‐point scale: motherese (high‐pitched talk directed to the simulator), neutral talk (regular adult talk directed to the simulator), talking to the self or the camera, singing, and no vocalization. Motherese was considered as the best indicator of sensitive behavior out of these types of vocalizations. *Adult touch* also included five options: affectionate touch (hugging, soothing, cradling, stroking, patting, that is, any touch that is meant to feel pleasant to the child or is meant to soothe the child), stimulating touch (tickling, “flying,” waving the simulator's limbs), functional touch (e.g., lifting the simulator's leg while changing the diaper), hold (holding the simulator without movement or further contact), and no touch. Affectionate touch was considered as the most sensitive behavior in the adult touch category. *Adult affect* was coded as either neutral, positive (smiling), or negative (frowning). Positive affect was considered as the best indicator of sensitive behavior in the adult affect category. Thus, each 5‐s interval received a score for each of the three categories.

Two coders, who were blind to the maternal status of the participants, double‐coded 12 videos (10% of the sample), with an interrater reliability (Cohen's *κ*) of .76. Two behavioral measures were chosen for the statistical analyses: the percentage of *affectionate touch* and the percentage of *motherese* across all 5‐s intervals. These two behavioral measures were chosen because they reflect sensitive caregiving behavior toward the simulator despite changes in the emotional state of the infant simulator and because earlier findings have demonstrated their association with oxytocin levels in mothers (Gordon et al., 2010). Adult affect was excluded from the analysis because of insufficient data: too often participants’ faces were not visible on the video, which prevented reliable evaluation of the participants’ affect.

#### 2.3.2 Oxytocin

Salivary oxytocin was analyzed with the Oxytocin ELISA kit (ENZO, cat.no ADI‐900‐153A). Salivary samples were purified with solid‐phase extraction strata‐X sorbent and 96‐well plate with 60 mg wells (Phenomenex 8E‐S100‐UGB). SPE columns were first revived with 1 ml of methanol. Methanol residues were washed with 1 ml of water. A total of 300 μl of 1.5% trichloroacetic acid (TFA) in water was added to 500 μl of saliva, stirred, and centrifuged at 6000 × *g* for 10 min. Samples were loaded into SPE columns, washed with 1.5 ml of 0.1% TFA, and eluted with 1 ml of acetonitrile (0.1% TFA, 80:20). Eluent was evaporated in vacuum centrifuge and samples were stored in a freezer (−20°C) until determination with the ENZO oxytocin ELISA kit according to the assay procedure. The coefficients of variation percent of intraassay and interassay of the method reported by the manufacturer were 12% and 16%, respectively. For oxytocin, we were able to analyze 64% of the first saliva samples and 63% of the second samples. The rest of the samples were either too low in oxytocin or there was not enough saliva for this assay.

#### 2.3.3 Cortisol

Salivary cortisol was analyzed with chemiluminescence immunoassay (LIA, IBL International, RE62011). Measuring range of the method is 0.43–88 nmol/L. The coefficients of variation percent of intraassay and interassay of the method were 5% and 7%, respectively. The analysis was successful for 94% of both of the two cortisol samples.

#### 2.3.4 Testosterone

Salivary testosterone was analyzed with enzyme immunoassay for the quantitative determination of free testosterone in saliva (EIA, IBL International, RE52631). Measuring range of the method is 10–900 pg/ml. The coefficients of variation percent for intraassay and interassay of the method were 6% and 9%, respectively. The analysis was successful for 97% of both of the two testosterone samples.

#### 2.3.5 Estradiol

Salivary estradiol was analyzed with luminescence immunoassay (IBL International, RE62141). Measuring range is 0.3–64 pg/ml. The coefficient of variation percent was 7.2%–13.3% for intraassay and 7.2%–14.8% for interassay. Analysis was successful for 80% of the first sample and 79% for the second sample. Estradiol was the last hormone to be analyzed from the saliva samples and unfortunately in some cases there was not enough saliva for the analysis (6% in the first sample and 9% in the second). Rest of the excluded estradiol samples were too low in measuring range.

#### 2.3.6 Positive and negative affects during the interaction

Participants’ positive and negative affects before and after the interaction were inquired with the PANAS questionnaire (Positive And Negative Affect Schedule; Watson et al., 1988), which consists of 20 words describing different emotions. Participants completed the schedule before the interaction with the simulator and again immediately after the interaction. Half of the words are positive (e.g., *enthusiastic*) and the other half negative (e.g., *nervous*). The participants were asked to assess how much they were experiencing the affect in question at that precise moment. The schedule is based on 5‐point Likert scale (from 1 = *very slightly or not at all* to 5 = *very much*). Sum scores of all positive and negative ratings indicate the positive and negative affect scales, respectively.

#### 2.3.7 Fertility motivation

The fertility motivation of the nulliparous women was assessed with the ABS (Brase & Brase, 2012). The Finnish version of the questionnaire consisted of 16 items instead of the original 34 items and included eight items of the *Positive Exposure* subscale and eight items of the *Negative Exposure* subscale. The items of the *Positive Exposure* subscale included positive experiences such as “*Looking after other people's babies makes me want to have a baby of my own*,” while the *Negative Exposure* subscale included items such as “*When I see an infant crying, I want to get as far away from the noise as possible*.” The items were coded in a 5‐point Likert scale (ranging from 1 = *Strongly disagree* to 5 = *Strongly agree*, with the Negative Exposure responses reversed) and an option for “*I don't know*.” A fertility motivation composite was calculated as a mean of the responses (Cronbach's *α* = .95). Items responded with “*I don't know*” were replaced with the mean of the participant's other values.

#### 2.3.8 Covariates

Age of the participant, time of day, cycle day, relationship duration, and years of education were initially considered as covariates based on earlier research. For mothers, the age of the infant and the time from the last breastfeeding were also included.

### 2.4 Statistical analyses

Video recordings were missing from three participants due to technical problems, but these participants’ hormonal data were included in the analysis. Two participants from the nulliparous group were excluded altogether due to technical problems with the infant simulator. Outliers in hormonal levels were examined and winsorized to 3 *SD*s (15 values in total). To achieve normality, oxytocin, estradiol, and testosterone values were square root transformed, whereas cortisol was log transformed. Hormonal reactivity scores were calculated as the difference between second and first saliva samples divided by the value of the first saliva sample. Reactivity scores were calculated from the winsorized untransformed values and used for correlation analysis. Missing data were estimated with multiple imputation implemented in IBM SPSS Statistics 27. In total, five imputation rounds were performed, and the pooled results are reported here. In total, 137 individual hormonal values were imputed (40 for T1 oxytocin, 41 for T2 oxytocin, 21 for T1 estradiol, 23 for T2 estradiol, five for each cortisol sample, and one for each testosterone sample).

Associations between the hormonal, behavioral, and background data were further investigated with Pearson correlation coefficients separately within the two groups (Table 3). In addition, to explore the influence of the categorically coded hormonal birth control use, we compared hormonal levels between women who did and did not use hormonal birth control separately in nulliparous and primiparous women using independent samples *t*‐tests. Two separate repeated‐measures ANOVAs were used to examine the change in participants’ positive and negative affects during the interaction task. Time (before and after the interaction) was the within‐subjects variable and parity was the grouping variable.

As can be observed from Table 1, the groups differed in some background variables. Age and the use of hormonal birth control were included as covariates in the main analysis. To answer the three main research questions, linear mixed models were conducted. One model was built for each hormone making it in total of four models. The −2‐log likelihood ratio scale was examined as a determinant of model fit. First, the repeated factor time (T1, T2) was added to the model, followed by within‐subjects covariates motherese, affectionate touch, age, and a dummy variable representing the use of hormonal birth control. Next, the between‐subjects factor parity was added. For the testosterone model, cortisol was also added as a covariate, to test whether the association between testosterone and maternal behavior was dependent on baseline cortisol level. Main effects of within‐ and between‐subjects variables were interpreted before adding any interaction terms to the models. Next, the interaction terms (parity × time, parity × motherese, parity × affectionate touch, time × motherese, time × affectionate touch, and the interaction terms of cortisol and the behavioral variables) were added. The final models are presented in the results.

Finally, to explore the relation of fertility motivation to the behavioral variables and hormonal reactivity within the nulliparous group, Pearson correlation coefficients between the ABS questionnaire scores and the behavioral and hormonal variables were calculated.

## 3 RESULTS

### 3.1 Preliminary analyses

#### 3.1.1 Descriptive statistics

As reported in Table 1, primiparous women were older than nulliparous women (*t*(115) = 5.99, *p* < .001, *d* = 1.12), their households had higher income (*χ* ^2^ = 33.98, *p* < .001), and their relationships had lasted longer (*t*(110) = −2.92, *p* = .004, *d* = 0.56). Nulliparous women used hormonal birth control more often than primiparous women (*χ* ^2^ = 21.90, *p* < .001). The two groups did not differ in the phase of menstrual cycle (*t*(72) = 1.73, *p* = .088, *d* = 0.43), years of education (*t*(110) = 1.74, *p* = .082, *d* = 0.33), or the time of day (*t*(115) = 1.86, *p* = .065, *d* = 0.35).

The task duration did not differ between primiparous and nulliparous women (*t*(111) = 1.37, *p* = .175, *d* = 0.25), and the simulator cried approximately the same time in both groups (*t*(111) = 0.13, *p* = .898, *d* = .02). Primiparous women expressed significantly more motherese in interaction with the simulator (*t*(111) = 4.18, *p* < .001, *d* = 0.79). There was no difference between primiparous and nulliparous women in the proportion of affectionate touch (*t*(111) = 1.14, *p* = .258, *d* = 0.21). The descriptive statistics and sample sizes for the winsorized untransformed values of the four hormones and the behavioral variables are presented in Table 2.

**TABLE 2:** Descriptive statistics of the hormonal levels and behavioral measures separately for nulliparous and primiparous women

	Nulliparous				Primiparous			
	*n*	*M* (*SD*)	Min	Max	*n*	*M* (*SD*)	Min	Max
T1								
Oxytocin (pg/ml)	44	12.68 (11.71)	0.06	52.49	31	22.39 (32.76)	1.73	135.28
Estradiol (pg/ml)	47	2.51 (1.86)	0.05	7.26	47	2.38 (2.02)	0.09	9.49
Testosterone(pg/ml)	61	25.75 (15.64)	1.01	76.92	53	23.74 (11.85)	6.61	67.19
Cortisol (nmol/L)	57	4.07 (3.05)	1.18	16.72	53	5.33 (5.52)	0.67	27.97
T2								
Oxytocin (pg/ml)	43	19.06 (29.19)	0.20	139.11	31	11.56 (6.43)	1.25	29.47
Estradiol (pg/ml)	48	2.25 (1.91)	0.01	7.76	44	2.42 (1.84)	0.01	8.28
Testosterone (pg/ml)	60	24.13 (13.76)	3.53	62.37	54	22.91 (13.53)	4.45	72.42
Cortisol (nmol/L)	57	3.73 (3.07)	0.90	14.74	53	4.28 (3.24)	0.32	15.18
Affectionate touch	59	37%	5%	75%	54	40%	7%	69%
Motherese*	59	48%	0%	99%	54	72%	0 %	99%

*Note*: Winsorized, untransformed, nonimputed data for the four hormones.

* *p* < .001.

Nulliparous women not using hormonal birth control had higher baseline testosterone levels than nulliparous women who did use hormonal birth control (*t*(59) = 2.53, *p* = .011, *d* = 0.67). Other hormonal baseline levels or reactivity did not differ as a function of hormonal contraceptive use in either of the two groups. Within the primiparous group, breastfeeding and non‐breastfeeding women did not differ in their baseline hormonal levels (Oxytocin: *t*(52) = −0.64, *p* = .524, *d* = 0.23; Estradiol: *t*(52) = 1.31, *p* = .191, *d* = 0.50; Testosterone: *t*(52) = −0.53, *p* = .598, *d* = 0.18; Cortisol: *t*(52) = 0.90, *p* = .369, *d* = 0.32).

In nulliparous women, the covariates (age, educational years, relationship length, menstrual phase, duration of the simulator crying, or time of day) did not correlate with any hormonal baselines or reactivity, or with behavior toward the infant simulator. The correlation coefficients between the study variables for nulliparous women are presented in Table 3 (below the diagonal).

**TABLE 3:** Correlation coefficients between study variables

		1	2	3	4	5	6	7	8	9	10	11	12	13	14	15	16	17	18	19
1	Oxytocin baseline	–	–.13	.17	–.17	.17	.40*	.06	–.03	.10	–.13	–.04	.07	.09	.23	–.09	–.08		–.01	.39**
2	Oxytocin reactivity	.20	–	–.06	.07	–.06	–.09	–.05	–.03	–.03	–.01	.00	.00	–.08	–.07	–.03	.04		.06	–.03
3	Estradiol baseline	.21	–.15	–	–.19	.04	–.02	.31*	–.05	–.12	–.17	.02	.19	.10	.18	–.10	–.26		–.05	–.10
4	Estradiol reactivity	–.23	.22	–.36	–	.04	–.00	–.07	.11	–.05	–.10	.00	–.04	–.15	–.12	.05	–.25		.00	–.05
5	Testosterone baseline	.04	.25	.16	.05	–	–.13	.22	–.01	–.19	–.36**	.04	–.11	–.03	–.00	.23	.13		–.13	.08
6	Testosterone reactivity	–.06	–.07	–.06	.05	–.38**	–	–.04	–.13	.25	.01	–.08	.17	.15	.23	–.31*	.03		.09	.46**
7	Cortisol baseline	.12	–.24	.20	–.23	–.08	–.19	–	–.22	–.04	–.33*	–.07	–.04	–.00	.14	–.03	.04		–.02	.07
8	Cortisol reactivity	.05	–.08	–.17	.24	–.10	.21	–.07	–	–.02	.04	.05	–.11	.19	–.24	.10	–.09		.04	–.08
9	Motherese	–.21	.02	–.05	.00	–.30*	.07	.18	.03	–	.27*	.18	.23	.28	.34*	–.24	.15		–.06	.05
10	Affectionate touch	–.18	–.08	.04	.07	–.17	.05	.13	.01	.28*	–	.22	.11	.15	.08	.08	.07		–.19	–.02
11	Duration of simulator crying	.35	–.12	.19	–.08	.25	–.16	–.09	–.09	.02	.02	–	.18	.00	.07	–.13	.20		–.14	–.13
12	Age	.17	.05	–.06	–.04	.06	.19	.05	.08	.03	–.09	.06	–	.43**	.42**	–.22	–.00		.29*	.13
13	Relationship duration	.23	.01	–.15	.09	.23	.03	–.05	.12	–.18	–.22	.08	.38**	–	.36*	.04	.01		.12	.33*
14	Educational years	.09	.17	.12	–.03	.09	–.07	–.00	.05	.22	–.14	.00	.57***	.16	–	–.00	.10		.02	.27
15	Time of day	–.05	.16	–.02	.04	.11	.13	–.14	.08	–.12	–.09	–.11	.27*	.14	.07	–	–.15		–.10	–.13
16	Menstrual phase	.06	.03	.13	.11	–.05	.11	–.04	.29	.05	.02	.23	–.32*	–.09	–.28	.05	–		–.17	–.11
17	Fertility motivation	–.10	–.19	–.23	–.03	–.40***	.10	–.02	–.02	.47***	.15	–.07	–.17	–.18	–.08	–.19	.09	–		
18	Age of infant																		–	.35*
19	Time from breastfeeding																			–

*Note*: Data for nulliparous women are shown below the diagonal and for primiparous women above the diagonal.

* *p* < .05 (two‐tailed)

** *p* < .01 (two‐tailed)

*** *p* < .001 (two‐tailed).

In primiparous women, time from last breastfeeding was positively correlated with baseline oxytocin (T1: *r* = .39, *p* = .01) and testosterone reactivity (*r* = .46, *p* = .002). Time of day was inversely associated with testosterone reactivity (*r* = –.31, *p* = .041). Educational years were associated with the use of motherese (*r* = .34, *p* = .017). Other covariates (relationship duration, menstrual phase, age, duration of simulator crying, or own infant's age) were not associated with any of the hormonal baseline levels, hormonal reactivity, or behavior toward the infant simulator within the primiparous group. Correlation coefficients for primiparous women are presented in Table 3 (above the diagonal).

#### 3.1.2 Positive and negative affect during the interaction

A repeated‐measures ANOVA showed a significant main effect of time on positive affect (*F*(1, 114) = 25.19, *p* < .001, $\eta_{p}^{2}\;$ = .18). Positive affect increased from before to after the interaction with the simulator (T1: *M* = 29.15, T2: *M* = 31.07). In addition, there was a significant main effect of parity (*F*(1, 114) = 5.78, *p* = .018, $\eta_{p}^{2}$ = .05), indicating that primiparous women experienced more positive affect across time than nulliparous women (T1: nulliparous *M* = 27.92, primiparous *M* = 30.54; T2: nulliparous *M* = 29.89, primiparous *M* = 32.41). There was no interaction between parity and time (*F*(1, 114) = 0.02, *p* = .899, $\eta_{p}^{2}$ = .00). There was a main effect of time on negative affect (*F*(1, 114) = 32.37, *p* < .001, $\eta_{p}^{2}$ = .22): Negative affect decreased from before to after the interaction (T1: *M* = 15.17, T2: *M* = 13.33). There was no main effect of parity on negative affect (*F*(1, 114) = 3.43, *p* = .067, $\eta_{p}^{2}$ = .03) nor an interaction between parity and time (*F*(1, 114) = 0.35, *p* = .558, $\eta_{p}^{2}$ = .00).

### 3.2 Hormonal reactivity and behavior towards the infant simulator

The linear mixed models for all four hormones are presented in Table 4.

**TABLE 4:** Linear mixed models

Models	ICC	Estimate	*SE*	95% CI	*t*	*p*
Oxytocin	.84					
Intercept		2.76	1.49	[−0.18, 5.70]	1.85	.066
Time		−0.04	0.14	[−0.32, 0.25]	−0.25	.807
Parity		−0.24	0.40	[−1.02, 0.54]	−0.61	.543
Motherese		−0.33	0.55	[−1.41, 0.76]	−0.59	.555
Affectionate touch		−1.83	1.08	[−3.98, 0.33]	−1.69	.095
Age		0.08	0.05	[−0.02, 0.19]	1.52	.131
Hormonal birth control^a^		−0.36	0.40	[−1.15, 0.44]	−0.89	.375
Parity × time		−0.48	0.28	[−1.03, 0.08]	−1.70	.091
Parity × motherese		0.82	1.06	[−1.28, 2.91]	0.77	.443
Parity × affectionate touch		−1.49	2.13	[−5.69, 2.71]	−0.70	.485
Time × motherese		−0.13	0.47	[−1.01, 0.80]	−0.28	.784
Time × affectionate touch		0.49	0.89	[−1.23, 2.26]	0.55	.586
Estradiol	.68					
Intercept		1.77	0.48	[0.83, 2.71]	3.68	.000
Time		−0.5	0.05	[−0.15, 0.05]	−0.93	.351
Parity		−0.05	0.14	[−0.32, 0.22]	−0.37	.712
Motherese		−0.13	0.20	[−0.52, 0.26]	−0.66	.511
Affectionate touch		−0.14	0.34	[−0.81, 0.54]	−0.40	.687
Age		0.00	0.02	[−0.03, 0.03]	0.04	.967
Hormonal birth control		−0.21	0.12	[−0.45, 0.03]	−1.73	.08
Parity × time		0.17	0.13	[−0.09, 0.43]	1.35	.185
Parity × motherese		−0.05	0.35	[−0.73, 0.63]	−0.14	.888
Parity × affectionate touch		−1.04	0.68	[−2.38, 0.30]	−1.53	.127
Time × motherese		−0.16	0.21	[−0.60, 0.28]	−0.74	.465
Time × affectionate touch		0.06	0.32	[−0.57, 0.68]	0.18	.861
Testosterone	.82					
Intercept		5.81	1.14	[3.57, 8.04]	5.10	.000
Time		−0.12	0.08	[−0.27, 0.03]	−1.60	.110
Parity		−0.11	0.31	[−0.71, 0.50]	−0.36	.721
Motherese		−0.60	0.42	[−1.43, 0.23]	−1.42	.157
Affectionate touch		−1.69	0.79	[−3.23, −0.15]	−2.14	.032
Age		0.01	0.04	[−0.06, 0.09]	0.34	.732
Hormonal birth control		−0.35	0.29	[−0.91, 0.21]	−1.22	.222
Cortisol		0.08	0.44	[−0.79, 0.96]	0.18	.856
Parity × time		−0.15	0.16	[−0.47, 0.18]	−0.88	.378
Parity × motherese		0.97	0.83	[−0.65, 2.30]	1.17	.241
Parity × affectionate touch		−1.89	1.74	[−5.29, 1.51]	−1.01	.276
Time × motherese		0.53	0.26	[0.02, 1.04]	2.03	.043
Time × affectionate touch		−0.32	0.51	[−1.30, 0.65]	−0.65	.514
Cortisol × Parity		1.30	0.90	[−0.46, 2.60]	−1.45	.148
Cortisol × Motherese		−0.14	1.37	[−2.82, 2.54]	−0.10	.919
Cortisol × Affectionate touch		2.17	2.79	[−3.38, 7.72]	0.78	.438
Cortisol	.21					
Intercept		0.47	0.26	[−0.03, 0.98]	1.86	.063
Time		−0.06	0.02	[−0.10, −0.02]	−3.04	.002
Parity		−0.05	0.07	[−0.09, 0.19]	0.67	.502
Motherese		0.09	0.10	[−0.11, 0.26]	0.81	.416
Affectionate touch		−0.18	0.18	[−0.53, 0.17]	−1.02	.310
Age		0.00	0.01	[−0.01, 0.02]	0.37	.710
Hormonal birth control		0.02	0.07	[−0.11, 0.15]	0.29	.775
Parity × time		−0.01	0.06	[−0.13, 0.13]	−0.21	.835
Parity × motherese		−0.07	0.18	[−0.42, 0.29]	−0.37	.712
Parity × affectionate touch		−0.88	0.38	[−1.63, −0.13]	−2.32	.021
Time × motherese		0.02	0.07	[−0.12, 0.15]	0.21	.832
Time × affectionate touch		0.14	0.14	[−0.12, 0.41]	1.05	.292

Abbreviation: CI, confidence interval; ICC, intraclass correlation coefficient.

^a^ Dummy variable (0 = not using hormonal birth control, 1 = using hormonal birth control).

#### 3.2.1 Oxytocin

Although mean values of the original data (Table 2) point to increased oxytocin levels following the infant simulator in nulliparous females and decreased levels in primiparous females, no significant main effects of time or parity, or interactions between parity and time were found in the linear mixed model on the imputed dataset. Neither motherese nor affectionate touch showed significant main effects or interaction with parity or time.

#### 3.2.2 Estradiol

For estradiol, there were no significant main effects of time or parity nor were there interactions between parity and time. Neither motherese nor affectionate touch had significant main effects or interaction with parity or time.

#### 3.2.3 Testosterone

**FIGURE 2:**
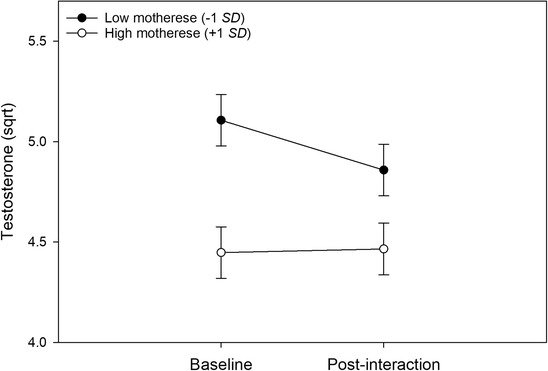
Regression lines representing the association between time and testosterone levels at low and high levels of motherese (± 1 *SD*)

For testosterone, there was a main effect of affectionate touch (*p* = .032). More affectionate touch was associated with lower overall testosterone levels across the two measurement points. There were no significant main effects of parity or motherese. Time showed a significant interaction with motherese (*p* = .043). To illustrate this interaction, standardized simple slopes were plotted for one standard deviation below and above the mean for motherese (Figure 2). The coefficients were in different directions signaling interaction. At low levels of motherese, testosterone levels were decreasing (*β* = −0.28, *p* = .346), whereas in high levels of motherese testosterone levels were slightly increasing (*β* = 0.153, *p* = .598).

Other interactions were not significant. To test for effects related to the dual hormone hypothesis, baseline cortisol was also included in the testosterone model. However, no interactions with cortisol emerged.

#### 3.2.4 Cortisol

**FIGURE 3:**
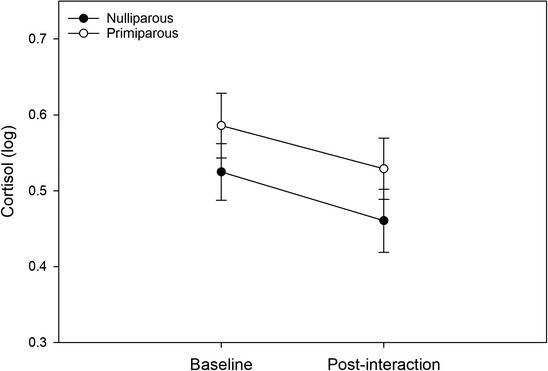
Cortisol reactivity to the infant simulator in primiparous and nulliparous women

**FIGURE 4:**
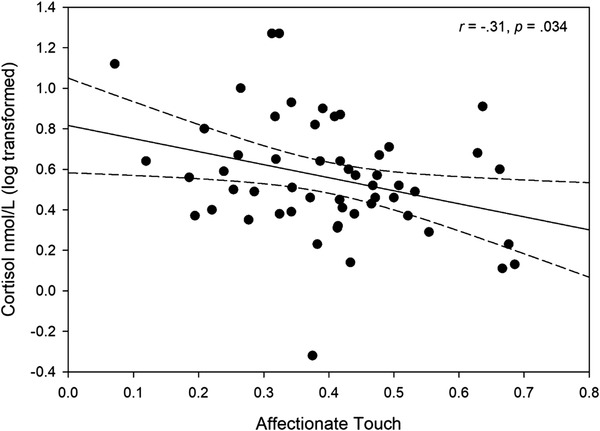
Correlation between overall cortisol levels and affectionate touch in primiparous women

Time had a significant effect on cortisol (*p* = .002), with cortisol levels showing an overall decrease from T1 to T2 (*t*(114) = 2.84, *p* = .005, *d* = 0.28) as can be observed from Figure 3. There was also a significant interaction between parity and affectionate touch (*p* = .021). We examined this interaction with additional linear mixed models conducted separately for both groups. For primiparous women, we found a significant main effect of affectionate touch on cortisol (*p* = .035), as more affectionate touch was associated with lower overall cortisol levels across the two measurement points (Figure 4). For nulliparous women, there was no significant main effect of affectionate touch on cortisol levels (*p* = .312).

### 3.3 Fertility motivation and its associations with behavior and hormones in nulliparous women

In nulliparous women, fertility motivation scores were positively associated with the amount of motherese used with the infant simulator (*r* = .47, *p* = .001) but not with affectionate touch. Fertility motivation was inversely correlated with baseline testosterone (T1: *r* = –.40, *p* = .001). The correlation coefficients are presented in Table 3 (below the diagonal).

## 4 DISCUSSION

The primary aims of the present study were to explore potential differences in salivary hormonal reactivity and their associations with behavioral responses in a simulated caretaking situation in primiparous and nulliparous females. The participants spent 6 min taking care of an infant simulator in a laboratory setting. The simulator made sounds ranging from crying to laughter mimicking a real infant and the participants were instructed to interact with the simulator as with a real infant. Salivary levels of oxytocin, cortisol, estradiol, and testosterone were measured before and after the situation to examine hormonal changes in response to exposure to the infant simulator, and indicators of sensitive caregiving behavior (affectionate touch and motherese vocalizations) were coded.

Cortisol levels decreased in response to the infant simulator in the whole sample. In line with the PANAS scores showing increased positive affect and decreased negative affect following interaction with the infant simulator, decreased cortisol levels suggest that the situation was not stress‐evoking and nulliparous and primiparous women were similar in their cortisol reactivity toward the infant simulator. Decreasing cortisol levels have also been observed in mothers when they take care of their own infant (Bos et al., 2018). Thus, the infant simulator appears to produce similar cortisol reactivity compared to interaction with a real infant. The decreasing cortisol levels in this study were opposite to earlier results with pregnant women in Bos et al. (2018), which suggests that during pregnancy cortisol reactivity may differ from other life situations.

We also observed an interaction between the amount of affectionate touch and parity in overall cortisol levels. In primiparous women, lower cortisol levels across the measurement points were associated with more affectionate touch, whereas in nulliparous women no association between cortisol levels and affectionate touch was found. The same difference was evident from the correlation coefficients (Table 3). This finding is novel and suggests that the association between cortisol and affectionate touch might be different in primiparous and nulliparous women. Earlier studies have not compared primiparous and nulliparous women in this regard, but our finding of an inverse relation between cortisol levels and affectionate touch in mothers is in line with earlier research suggesting that lower baseline cortisol levels are associated with higher maternal sensitivity (Finegood et al., 2016; Gonzalez et al., 2012) and less intrusive parenting behavior (Mills‐Koonce et al., 2009). In addition, mothers’ sensitivity toward an infant simulator has been observed to correlate with their sensitivity toward their own infant (Bakermans‐Kranenburg et al., 2015). Together with the current study, those findings support the use of the infant simulator as a valid method for investigating individual differences in caretaking behavior and suggest that mothers differ from nonmothers regarding the association between cortisol and caretaking behavior also during naturalistic caretaking situations. In the current study, mothers also used more motherese with the simulator than nonmothers, suggesting that they were behaving with the simulator as with their own infant. However, we did not control for the reported seriousness or reality value (see Bos et al., 2018; Voorthuis et al., 2013) of the interaction with the simulator, which is necessary in the future studies.

There was a negative association between testosterone levels and the amount of affectionate touch when taking care of the simulator. This is in line with the earlier research with male samples (Bos et al., 2018; Meijer et al., 2019; Weisman et al., 2014) and thus indicates that testosterone might have similar associations with caretaking behavior in females and males. We also observed an interaction between time and motherese for testosterone levels: at low levels of motherese, testosterone levels decreased in response to the interaction with the infant doll, whereas at high levels of motherese testosterone levels increased during the interaction with the doll. However, when studying mothers, Bos et al. (2018) did not find any associations between prenatal testosterone levels and caretaking behavior toward an infant simulator, nor did they find any associations between postnatal testosterone levels and caretaking behavior with their own infant. Comparisons between the current study and Bos et al. (2018) should be made cautiously, however, as different interactive behaviors were observed in these studies. While Bos et al. (2018) used an overall index of maternal sensitivity, in our study we focused on more focal aspects of behavior (motherese and affectionate touch). It is therefore possible that the differences in the results of these studies may depend on the different coding schemes. In the future, it will be important to evaluate the feasibility of different coding schemes within the same sample.

In this study, primiparous and nulliparous women did not show different associations between testosterone and caretaking behavior in the main analysis. In addition, we found no evidence of testosterone reactivity to the infant simulator (i.e., a main effect of time) in the full sample nor a difference in reactivity between primiparous and nulliparous women. In earlier studies, testosterone levels have been found to increase in pregnant women taking care of an infant simulator (Bos et al., 2018), whereas in nulliparous women testosterone levels have been found to decrease (Voorthuis et al., 2019). Thus, our findings differ especially from those of Voorthuis et al. (2019) regarding the nulliparous group. However, the infant simulator paradigm in our study differs in important ways from those of earlier studies that have included longer periods of crying and the lack of possibilities to sooth the infant simulator. This may have triggered stronger testosterone reactivity. Furthermore, we did not find cortisol to moderate the association between testosterone and behavior. One reason for this might be due to the all‐female sample in our study. Earlier human and animal studies have had either mixed‐sex or all‐male samples and it is possible that the moderating effect of cortisol on testosterone levels is more evident in males than females.

The two groups did not show oxytocin reactivity toward the infant simulator, nor did they differ in their oxytocin reactivity toward the simulator. This was contradictory to our expectations and results from studies investigating parental oxytocin reactivity in response to interaction with their infant (e.g., Feldman et al., 2010). However, in our study, the amount of oxytocin was below the manufacturer's limit in 48% of the sample and 36% of the saliva samples were unanalyzable due to the low levels of oxytocin or insufficient amount of saliva, which means that oxytocin data were based on imputed values to a greater extent than was the case for other hormones. Therefore, the oxytocin results should be evaluated with caution. The wide variability of reported oxytocin levels across the literature has resulted in criticism toward measuring oxytocin from saliva (Horvat‐Gordon, et al., 2005; McCullough et al., 2013). Many of the previous studies that reported highly variable oxytocin levels were missing the extraction step of the analysis. In this study, the oxytocin samples were extracted before the assay, which, as a downside, partially explains the attrition in the oxytocin data. Furthermore, similar to earlier studies, in our sample baseline oxytocin levels were correlated with the time since the last breastfeeding in mothers. This indicates that oxytocin levels as measured in the present study reflect true variation as in earlier studies oxytocin levels have been found to start to increase after breastfeeding reaching their peak just before the next feeding (Carter et al., 2007; de Jong et al., 2015; White‐Traut et al., 2009). It is noteworthy that a considerable proportion of the literature documenting associations between peripheral oxytocin and parenting behaviors is based on data from a single laboratory (see Grumi et al., 2021). Therefore, it is vital to replicate and extend such associations with greater diversity of samples and methods.

Compared to other hormones in this study, there is very little research on the relation of estradiol to caretaking in humans. In our study, estradiol levels did not change in response to interaction with the simulator, nor were they associated with caretaking behavior. The few earlier studies linking estradiol levels to parenting or relationship outcomes (Edelstein et al., 2017; Glynn et al., 2016) have studied estradiol levels during pregnancy. As estradiol levels increase during pregnancy and decrease rapidly after giving birth (Fleming, Ruble, et al., 1997), associations between estradiol and caretaking behavior may be more prominent during the prenatal period. In addition, associations between estradiol and behavior might depend on individual progesterone or testosterone levels, which have been found to be important in earlier studies. For example, smaller decline in the estradiol to progesterone ratio during pregnancy has been associated with higher postpartum feelings of attachment toward the infant (Fleming, Ruble, et al., 1997), and in fathers high testosterone levels combined with high, but not low, estradiol levels have been associated with lower sensitivity (Bakermans‐Kranenburg et al., 2022). In the future, it is relevant to also measure progesterone, which might have a moderating effect on estradiol reactivity.

In nulliparous women, higher fertility motivation was associated with more motherese directed to the simulator. This finding is novel and indicates that women's positive feelings toward infants and motivation to become a mother affect their caretaking behavior in a positive way. In addition to behavior, fertility motivation was negatively associated with testosterone levels. In line with the Challenge hypothesis (Archer, 2006), the inverse relation between fertility motivation and testosterone may suggest that, similarly to men, preparation to parenthood is associated with declining testosterone levels also in women. Another possible explanation for the negative association between testosterone and fertility motivation could be that women who have lower testosterone levels in general have more positive views on babies. This would also be in line with earlier results showing higher testosterone levels to be associated with lower self‐rated reproductive ambition (Deady et al., 2006). Fertility motivation or “baby fever,” although being a popular subject on the media, has not yet been studied extensively. It is unclear whether fertility motivation predicts future pregnancy in women. In addition, the potential associations between fertility motivation and hormones and caretaking behavior in men are an important target for future studies.

Together with earlier studies using the infant simulator (Bos et al., 2018; Voorthuis et al., 2019), this study supports the use of the infant simulator in comparing mothers and nonmothers: the infant simulator elicited hormonal reactivity in both mothers and nonmothers, and the two groups showed similar and partially different patterns of associations between hormonal levels and caregiving behavior. However, our results are preliminary at best and require replication in independent samples and greater variability of caregiving behaviors in the future. In addition, it remains unclear when during the transition to parenthood the potential differences in hormonal reactivity and caretaking behavior begin to emerge. Longitudinal research designs would be important to determine whether the differential responses emerge in mothers due to the biological changes associated with pregnancy or whether they are induced by caretaking experiences with their infants. Comparing biological and adoptive parents in a simulated caretaking situation might reveal an answer to this question. In addition, the impact of hormones on different aspects of caretaking behavior may be indirect and operate through motivational processes that may affect parental behavior. For example, oxytocin has been linked to approach motivation (MacDonald & MacDonald, 2010; Soriano et al., 2020) and activity of the reward system of the brain (MacDonald & MacDonald, 2010). The heightened motivation toward babies could thus promote infant‐oriented behavior such as the use of motherese. In future studies, it will be important to investigate the role of motivation toward babies for parental behavior in mothers and nulliparous women in greater detail.

## CONFLICT OF INTEREST

The authors declare no conflict of interest.

## Data Availability

Data are available on Open Science Framework: https://osf.io/8rh26/.
